# COMBINING MACHINE LEARNING WITH AUTOMATED NEMATODE LIFESPAN ANALYSIS TO IDENTIFY MODIFIERS OF ALZHEIMER’S DISEASE

**DOI:** 10.1093/geroni/igz038.361

**Published:** 2019-11-08

**Authors:** Joshua Russell, Matt Kaeberlein

**Affiliations:** University of Washington, Seattle, Washington, United States

## Abstract

Here we present new computational and experimental methods to leverage the gene expression and neuropathology data collected from several large-scale studies of Alzheimer’s disease . These data sets include diverse data types, including transcriptomics, neuropathology phenotypes such as quantification of amyloid beta plaques and tau tangles in different brain regions, as well as assessments of dementia prior to death. This meta-analysis is a complex undertaking because the available data are from different studies and/or brain regions involving study-specific confounders and/or region-specific biological processes. We have therefore taken neural network and probabilistic computational approaches that reduce the data dimensionality, allowing statistical comparison across all brain samples. These approaches identify gene expression changes that are significantly associated with clinical and neuropathological assessment of Alzheimer’s disease. We then conduct in vivo validation of the genes through genetic screening of C. elegans models of Alzheimer's disease utilizing our automated robotic lifespan analysis platform. This approach allows for the greater leverage of existing Alzheimer’s disease biobank data to identify deep genetic signatures that could help identify new clinical gene-expression markers and pharmacological targets for Alzheimer’s disease.

